# Antibiotic Resistance of *Enterococcus* Species in Ornamental Animal Feed

**DOI:** 10.3390/ani13111761

**Published:** 2023-05-26

**Authors:** Rúben Soares, Carla Miranda, Sandra Cunha, Luís Ferreira, Ângela Martins, Gilberto Igrejas, Patrícia Poeta

**Affiliations:** 1 Microbiology and Antibiotic Resistance Team (MicroART), Department of Veterinary Sciences, University of Trás-os-Montes and Alto Douro, 5000-801 Vila Real, Portugal; al70477@alunos.utad.pt (R.S.); al62192@utad.eu (S.C.); al65041@utad.eu (L.F.); ppoeta@utad.pt (P.P.); 2 Associated Laboratory for Green Chemistry (LAQV-REQUIMTE), University NOVA of Lisbon, 1099-085 Caparica, Portugal; gigrejas@utad.pt; 3 Toxicology Research Unit (TOXRUN), University Institute of Health Sciences, Advanced Polytechnic and University Cooperative (IUCS-CESPU), 4585-116 Gandra, Portugal; 4 Department of Zootechnics, University of Trás-os-Montes and Alto Douro, 5000-801 Vila Real, Portugal; angela@utad.pt; 5 Veterinary and Animal Research Centre (CECAV), University of Trás-os-Montes and Alto Douro, 5000-801 Vila Real, Portugal; 6 Associate Laboratory for Animal and Veterinary Science (AL4AnimalS), University of Trás-os-Montes and Alto Douro, 5000-801 Vila Real, Portugal; 7 Department of Genetics and Biotechnology, University of Trás-os-Montes and Alto Douro, 5000-801 Vila Real, Portugal; 8 Functional Genomics and Proteomics Unit, University of Trás-os-Montes and Alto Douro, 5000-801 Vila Real, Portugal

**Keywords:** antibiotic resistance, *Enterococcus*, feed, ornamental animals, virulence genes

## Abstract

**Simple Summary:**

Numerous studies have already reported the presence of different antibiotic-resistant *Enterococcus* species in food-producing animals, animal products and pet food. However, studies specifically evaluating antimicrobial resistance in *Enterococcus* spp. in ornamental animals and their food are scarce. Therefore, this study aimed to identify *Enterococcus* spp. and their antibiotic-resistant patterns in ornamental animal feed, as this could lead to the spread of antimicrobial resistance to humans due to their close contact with these animals.

**Abstract:**

*Enterococcus* is a bacterial genus that is strongly associated with nosocomial infections and has a high capacity to transfer and acquire resistance genes. In this study, the main objective was to evaluate the presence of *Enterococcus* species in ornamental animal feed and characterize their antimicrobial resistance and virulence factors. Antimicrobial susceptibility was determined using 14 antimicrobial agents by the disk diffusion method, complemented by genotypic analysis to identify *Enterococcus* species and the presence of 14 antimicrobial resistance and 10 virulence genes. From 57 samples of ornamental animal feed, 103 *Enterococcus* isolates were recovered from 15 bird, 9 fish and 4 reptile feed samples. *Enterococcus* isolates were highly resistance to rifampicin (78%) and erythromycin (48%), and 48% of isolates were classified as multidrug-resistant. *Enterococcus faecalis* (36.7%) and *E. faecium* (31.7%) were the species most frequently identified. Most isolates carried the resistance genes *erm*B (57%) and *tet*L (52%) and the virulence genes, *cyl*L (52%) and *esp* (40%). *Enterococcus gallinarum* was the species with the highest number of multidrug-resistant isolates (50%) and virulence genes (80%). These results highlight the high levels of antibiotic-resistant *Enterococcus* spp. present in ornamental animal feed and the growing interaction of these animals with humans as a public health concern.

## 1. Introduction

*Enterococcus* is a bacterial genus predominantly found in the intestinal microbiota of humans and animals [1] and is sporadically detected in the oral cavity, upper respiratory system and vaginal tract [2]. These bacteria are often found in food, plants, water and soil due to fecal contamination [3]. Currently, several *Enterococcus* species are known, such as *E. avium*, *E. asini*, *E. casseliflavus*, *E. cecorum*, *E. dispar*, *E. durans*, *E. faecalis*, *E. faecium* and *E. gallinarum* [4,5]. However, *E. faecalis* and *E. faecium* are the two most isolated and studied species. This group of microorganisms is strongly associated with nosocomial infections, such as endocarditis, septicemia, wound and urinary tract infections and meningitis [6,7,8,9]. Infections caused by *Enterococcus* spp. have identified isolates with resistance to several antimicrobial classes, including aminoglycosides, β-lactams, glycopeptides, streptogramins and lincosamides. Cases of multidrug resistance (resistance to ≥3 antimicrobial classes) have been reported [10]. In addition, *Enterococcus* demonstrates an enormous capacity to transfer and acquire resistance genes, produce biofilms and virulence factors and facilitate the induction of inflammatory processes and antimicrobial resistance [11,12]. Furthermore, this high capacity to antibiotic resistance of *Enterococcus* can be acquired through horizontal exchange of genetic material, carriers’ resistance determinants or sporadic mutation of intrinsic genes, or it can be intrinsic to the species. For instance, *E. faecalis* is intrinsically resistant to several antibiotics like streptogramins and lincosamides [10].

Antimicrobial resistance represents a serious risk to the environment and especially to human and animal health [13], apart from the economic consequences resulting from the increase in healthcare costs [14]. This silent pandemic has been aggravated by the inappropriate and overuse of antibiotics in human and animal medicine [15]. The contact between humans and animals is increasing, either with animals produced for human consumption or with companion animals, including ornamental animals. This proximity may have implications for the epidemiology of these microorganisms and public health. Animals are described as potential reservoirs of antimicrobial resistance, contributing to the dissemination of resistance genes in the environment and/or transfer to other animals and humans. Several studies have already demonstrated the presence of antibiotic-resistant *Enterococcus* spp., mainly in food-producing animals and animal foods [16,17,18]. Regarding ornamental animals, limited data are available where isolates of *Enterococcus* spp. have been researched, although the interaction of these animals with humans has increased in recent years. Ahmed et al. [19] highlighted that certain multidrug-resistant and virulent zoonotic bacteria carried by ornamental birds can pose a risk to other birds and to their owners or caretakers [19].

Thus, the objectives of this study were to evaluate the presence of different *Enterococcus* species in samples of food supplied to ornamental animals and characterize their phenotypic and genotypic antibiotic resistance and virulence genes. 

## 2. Materials and Methods

### 2.1. Isolates

A total of 57 samples of ornamental animal feed (birds, fish, mammals and reptiles) were obtained from a range of pet food supermarkets between February and December 2020. From each sample of feed, 2 g were collected, while 1 g was enriched in 5 mL of brain heart infusion broth (Liofilchem^®^ s.r.l., Roseto d. Abruzzi, Italy) at 37 °C for 12 h. *Enterococcus* putative isolates were obtained from plates with Slanetz–Bartley agar (Liofilchem^®^ s.r.l., Roseto d. Abruzzi, Italy), supplemented with vancomycin (4 μg mL^−1^), after a period of incubation at 37 °C for 24–48 h. Up to four typical colonies per sample with compatible enterococcal morphology were isolated or incubated in kanamycin aesculin azide agar (Liofilchem^®^ s.r.l., Roseto d. Abruzzi, Italy), and their identification was confirmed by standard biochemical tests like Gram staining, catalase test and growth in the presence of 6.5% NaCl [20]. The remaining samples were used to evaluate the total microbial level or viable bacterial growth present in each sample as described in the next point.

For species characterization and based on the origin of the sample and observation of different macroscopic morphology of colonies, 60 representative isolates of all obtained *Enterococcus,* confirmed by microbiological and biochemical methods, were selected for the detection of the following species: *E. faecalis (ddl _E. faecalis_*), *E. faecium (ddl _E. faecium_*), *E. gallinarum* (*van*C1) and *E. durans* (*mur*-2). DNA extraction was performed using the commercial GRS Genomic DNA Kit (GRISP Research Solutions, Porto, Portugal), following the manufacturer’s instructions. For each species, identification was performed by PCR assays using specific primers [21,22] and conditions, as previously described by Guerrero-Ramos et al. [1]. The results were visualized by agarose gel electrophoresis. Negative (water) and positive (collection of the University of Trás-os-Montes and Alto Douro) controls were used in all PCR assays [1].

### 2.2. Total Microbial Level

To determine the total microbial level, 1 g of each sample was diluted in sterile distilled water in the proportions 1:10, 1:100 and 1:1000. Then, 100 μL was transferred from each dilution and cultured in non-selective Plant Count Agar (PCA, Liofilchem^®^ s.r.l., Roseto d. Abruzzi, Italy), using an L Seeder. Subsequently, the plates were incubated at 37 °C for 24 h, and after this period, the results were recorded, and colonies from the plates with growth were counted and registered. The microorganism’s concentration of amount was expressed as colony-forming unit per gram (CFU/g) of feed.

### 2.3. Antimicrobial Susceptibility Testing

For all obtained microbiological isolates, antimicrobial susceptibility was carried out using 14 antimicrobial agents (Liofilchem^®^ s.r.l., Roseto d. Abruzzi, Italy), including ampicilin (10 μg), gentamicin (120 μg), erythromycin (15 μg), fosfomycin (200 μg), ciprofloxacin (5 μg), chloramphenicol (30 μg), linezolid (30 μg), nitrofurantoin (300 μg), quinupristin/ dalfopristin (15 μg), rifampicin (5 μg), streptomycin (300 μg), tetracycline (30 μg), teicoplanin (30 μg), and vancomycin (30 μg). The Kirby–Bauer disk diffusion method was used for testing, according to the Clinical and Laboratory Standards Institute (CLSI) standards [23].

Each *Enterococcus* isolate was inoculated onto plates with Mueller–Hinton II agar (Oxoid^®^, Basingstoke, UK) impregnated with various antibiotic disks in different concentrations as described above, at 0.5 McFarland standard. The plates were then incubated at 37 °C for 18–24 h [23], and the zone of inhibition formed around each disk was measured according to the CLSI guidelines [23] and registered as sensitive and resistant, including the intermediate.

### 2.4. Resistance and Virulence Genes

The presence of antibiotic resistance were tested in the extracted DNA, namely resistance genes for erythromicyn (*erm*A, *erm*B and *erm*C), tetracycline (*tet*L, *tet*M, *tet*K and *tet*O), quinupristin/dalfopristin (*vat*D and *vat*E), gentamicin (*aac*(6′)-*aph*(2″)), chloramphenicol (*cat*A), streptomycin (*ant*(6)-*Ia*) and vancomycin (*van*A and *van*B). 

For virulence factors, we tested for the presence of 10 genes, such as enterococcal surface protein (*esp*), accessory colonization factor (*ace*), gelatinase (*gel*E), aggregation substance (*agg*), regulator of the expression of *gel*E (*fsr*), pheromone determinant (*cpd*) and cytolysin (*cyl*A, *cyl*B, *cyl*M and *cyl*L). 

The results of the PCR assays, using specific primers [21,24,25,26,27,28,29] and conditions, as previously described by Guerrero-Ramos et al. [1], were visualized by agarose gel electrophoresis. Negative and positive controls were used in all PCR assays from the strain collection of the University of Trás-os-Montes and Alto Douro [1,20].

### 2.5. Statistical Analysis

The data were statistically analyzed using the SPSS 15^®^ software (SPSS Inc., Chicago, IL, USA). Univariate and quantitative analyses of discrete variables were performed for each variable through absolute and relative frequency measures.

For statistical analysis of association between variables, the chi-square (χ^2^) independence test was used. A probability level (*p*) < 0.05 was considered statistically significant in the association of variables.

## 3. Results

### 3.1. Total Microbial Level

**Figure 1:**
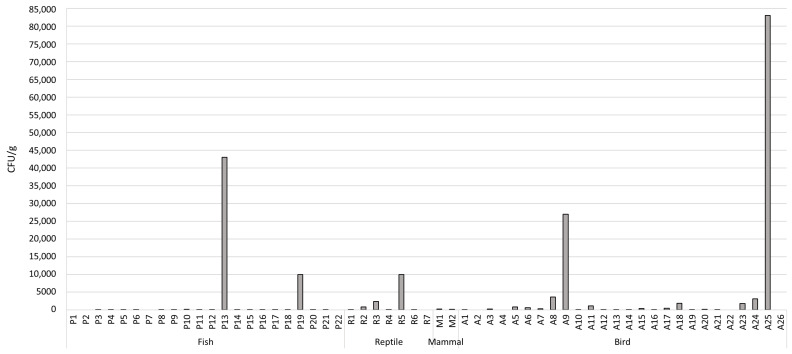
Total microbial level obtained in all ornamental feed samples analyzed in this study expressed as colony forming unit per gram (CFU/g) of feed.

From the 57 analyzed samples, 84.2% (*n* = 48) showed microbial growth in PCA, while 15.8% (*n* = 9) did not (Figure 1 and Table S1). The highest microbial level was observed in a bird food sample (sample ID: A25) and fish food sample (sample ID: P13), with 83,000 CFU/g and 43,000 CFU/g, respectively. The values of microbial growth ranged from 10 to 83,000 CFU/g for bird samples, 10 to 43,000 CFU/g for fish samples, 10 to 10,000 CFU/g for reptiles, and 200 to 260 CFU/g for mammal samples (Table S1).

### 3.2. Enterococcus Isolates/Species Identification

Microbiological and biochemical tests identified 103 *Enterococcus* isolates from the 28 of 57 feed samples. Of the 103 isolates, 56.3% (*n* = 58) were obtained from bird feed; 32% (*n* = 33) were obtained from fish feed, and 11.7% (*n* = 12) were obtained from reptile feed (Table 1). No isolates of presumptive *Enterococcus* were obtained in mammalian samples. 

Of the 60 selected enterococci for species confirmation by PCR assay, 56.7% (*n* = 34) were from bird samples; 31.6% (*n* = 19) were from fish, and 11.7% (*n* = 7) were from reptile samples. The following species were identified from these isolates: 36.7% (*n* = 22) of *E. faecalis*, 31.7% (*n* = 19) of *E. faecium*, 25% (*n* = 15) of *E. gallinarum* and 6.7% (*n* = 4) of *E. durans*.

### 3.3. Antimicrobial Susceptibility Characterization

Table 1 shows the phenotypic antibiotic resistance profile for the 103 *Enterococcus* isolates based on the antimicrobial classes and origin of animal feed samples. A high prevalence of resistance was observed for rifampicin (77.7%). In addition, resistance to erythromycin (48.5%), ciprofloxacin (37.9%), tetracycline (26.2%), linezolid (19.4%), nitrofurantion (18.4%) and fosfomycin (6.8%) was also observed. For the remaining tested antibiotics, the isolates showed a low level of resistance, such as chloramphenicol (3.9%), ampicillin (2.9%), vancomycin (2.9%) and teicoplanin (2.9%). No isolates showed resistance to streptomycin and gentamicin. As expected, all *Enterococcus* isolates were intrinsically resistant to quinupristin/dalfopristin (Table 1).

Clustering the different tested antibiotics by their classes, the results showed that 47.6% of the isolates were classified as multidrug-resistant (resistance to ≥ 3 antimicrobial classes), of which 17.5% were resistant to five or more classes; 6.8% were resistant to four classes, and 23.3% were resistant to three antimicrobial classes. The remaining 26.2% and 17.5% of enterococcal isolates showed resistance to 2 and 1 antimicrobial classes, respectively, and 8.7% of isolates did not show resistance to any antibiotic (Table S2).

### 3.4. Genotypic of Antibiotic Resistance and Virulence Genes

From 60 selected enterococcal isolates, the detection of the presence of antibiotic-resistant genes showed that the most prevalent resistance genes were *erm*B (56.7%), *tet*L (51.7%), *tet*M (36.7%) and *van*A (38.3%). On the other hand, the least identified resistance genes were *erm*A, *vat*D and *aac*(6″)-*aph*(2″). No amplification was observed for gene *tet*O in any of the isolates (Table 2).

The resistant gene to erythromicyn, *erm*B, was found in *E. faecalis* (18.2%), *E. faecium* (52.6%), *E. gallinarum* (86.7%) and all *E. durans* isolates. For tetracycline resistance genes in *E. faecalis*, 18.2% of the isolates contained the *tet*K gene, and another 18.2% contained the *tet*M gene, and only 4.5% of isolates showed the *tet*L gene individually. In addition, the combination *tet*M + *tet*L was detected in 13.6% of the isolates, and the combination *tet*K + *tet*M was found in 4.5% of the isolates of *E. faecalis*. In the case of *E. faecium*, 42.1% of the isolates showed only the *tet*L gene, and the *tet*K + *tet*L combination (26.3%) was also present. The combinations *tet*M + *tet*L, *tet*K + *tet*M, *tet*K + *tet*L and *tet*K + *tet*M + *tet*L were found in 21%, 10.5%, 10.5% and 15.8% of the *E. gallinarum* isolates, respectively. All *E. durans* isolates showed resistance to tetracycline, with 50% containing the *tet*M + *tet*L combination and the remaining 50% containing the combination *tet*K + *tet*M + *tet*L (Table S2).

Regarding the vancomycin-resistant isolates in *E. faecalis*, 4.5% showed the presence of the *van*A gene; 9% showed the presence of the *van*B gene, and 18.2% showed the *van*A + *van*B combination. In *E. faecium*, 26.3% of the isolates contained the *van*A gene, while 15.8% had the *van*B gene. The *van*A + *van*B combination was identified in one isolated. On the other hand, *E. gallinarum* demonstrated the presence of *van*A in 60% of its isolates, with only one isolate containing the *van*B gene and the other the combination *van*A + *van*B. Likewise, 50% of the *E. durans* isolates were positive for the presence of the *van*A gene.

In contrast to the *E. faecalis* and *E. faecium* species, most of the *E. gallinarum* and *E. durans* isolates showed the *ant*(6)-Ia gene (66.7% of *E. gallinarum* isolates and 100% of *E. durans* isolates) and the *cat*A gene (60% of *E. gallinarum* and 50% of *E. durans*).

The *vat*D and *vat*E genes, associated with resistance to quinupristine-dalfopristine, were found in 25% of isolates, and the *vat*E gene was the most prevalent in the species *E. faecalis*, *E. faecium* and *E. gallinarum*. In *E. durans*, 50% of the isolates contained the gene *vat*D, and 25% contained the *vat*E gene (Table 2).

A total of 46.7% (28/60) of *Enterococcus* isolates were genotypically classified as multidrug-resistant bacteria. *Enterococcus gallinarum*, followed by *E. faecium*, was the species with the most multidrug-resistant isolates, 50% and 22%, respectively.

Virulence genes were also identified, with a greater predominance of *cyl*L and *esp* genes, identified in 51.7% and 40% isolates, respectively. The *ace* and *fsr* genes were not found in any of the species under study (Table 3). In total, 18 isolates did not show any virulence gene. The species *E. gallinarum* presented the most virulence genes, while in *E. faecium*, the presence of these genes was residual.

The statistical analysis did not include the *E. durans* isolates due to their reduced number. The results demonstrated significant associations (*p* < 0.05) for the *erm*B, *ant*(6)-Ia, *van*A, *tet*M and *tet*L resistance genes. Thus, *E. faecalis* was more likely to be positive for *tet*M; *E. faecium* was more likely to be positive for *erm*B and *tet*L, and *E. gallinarum* was more likely to be positive for *erm*B, *ant*(6)-Ia, *van*A and *tet*L. For virulence factors, a significant association was observed for the *cyl*L gene. *E. gallinarum* was more likely to be positive for *cyl*L, demonstrating a higher probability of this species carrying the *cyl*L gene (Table 4).

Briefly, Table S2 comprises the phenotypic, genotypic and virulence profiles of each of the 60 isolates obtained in this study, as well as the feed origin. 

## 4. Discussion

Enterococci are natural inhabitants of the gastrointestinal tract of mammals, humans, feed and insects. Although enterococci were initially classified as commensal bacteria not pathogenic, they are currently responsible for the most common nosocomial infection in humans and animals [30]. A high level of multidrug resistance in human clinical *Enterococcus* strains was identified in Slovenia, with 73.3% of *E. faecium* and 29.6% of *E. faecalis* showing resistance [14]. The emergence of multidrug resistance in enterococcal species is a public health problem, limiting therapeutic options. However, information in the literature about resistance to antibiotics in ornamental animal feed is still very scarce. In our study, the presence of *Enterococcus* was identified in 28 of 57 ornamental animal feed samples. Recently, Dolka et al. [31] evaluated the presence of *Enterococcus* spp. in racing pigeons (*Columba livia* f. Domestica) and observed that almost all isolates, about 93.1%, were resistant to at least one antibiotic, with *E. faecalis* being one of the most frequently identified species in these birds. In that study, *Enterococcus* showed resistance more frequently to teicoplanin (73%) and erythromycin (75.2%). Another study [14] detected resistance to tetracycline (78.9%) and erythromycin (46.5%) in *E. faecalis* from retail red meat and human clinical samples. In the case of *E. faecium*, most clinical isolates were resistant to erythromycin (45%), followed by ciprofloxacin (41%) and ampicillin (41%). In comparison with Dolka et al. [31] and Golob et al. [14], our study also showed that *Enterococcus* is resistant to erythromycin (48.5%) but has a lower percentage of resistance to tetracycline (26.2%) and teicoplanin (2.9%). Another aspect worth reporting is the low resistance to vancomycin, as also described by Marinho [32].

With regard to pets, such as dogs and cats, Trościańczyk et al. [33] recorded results very similar to those mentioned above, with a high prevalence of resistance of *Enterococcus* to erythromycin (96%), ciprofloxacin (93%) and tetracycline (82%) and a significant number of isolates showing multidrug resistance (78%). In our study, none of the isolates showed resistance to gentamicin and streptomycin, similar to the study of Osman et al. [34]. Freitas et al. [35] reported that 54% of the 55 studied dog food samples contained *Enterococcus* spp., of which 31% were considered multidrug-resistant. However, the number of multidrug-resistant isolates is higher in our study when compared to Golob et al. [14] and Freitas et al. [35]. *E. faecalis* and *E. faecium* were the most frequently identified species, which is consistent with the results obtained in other studies, mainly in studies where *Enterococcus* spp. were isolated from source food animal [1,14]. *E. gallinarium* was the *Enterococcus* species that demonstrated a higher probability of carrying a variety of antibiotic-resistant genes based on statistical analysis.

In general, based on the results of this study, it can be concluded that the *erm*B gene is the most representative in erythromycin resistance in *Enterococcus* isolates, just as the *tet*L and *tet*M genes are described as the main genes that encode tetracycline resistance. The gene combinations *erm*B-*tet*M or *erm*B-*tet*M-*tet*L were present in 27% of the isolates, confirming the fact that the *erm*B gene is usually linked to the *tet*M gene in the mobile conjugative transposon *Tn1545*. Usually, tetracycline resistance genes are found on the same mobile unit as the genes that confer resistance to macrolides [36]. In our study, these combinations were predominant in the species *E. durans* and *E. gallinarum*. Alternatively, the gene *aac*(6′)-*aph*(2′′) that is normally associated with resistance to gentamicin was identified only in one *E. durans* isolate and two *E. gallinarum* isolates, which is consistent with the phenotypic results, where no isolate showed resistance to gentamicin. Freitas et al. [35] identified this gene in *E. faecalis* and *E. faecium* from raw dog food samples.

At the genotypic level, 20% of the *Enterococcus* spp. identified in this study carried the *cat*A gene, which is similar to the results obtained by Osman et al. [34] in Nile tilapia with streptococcosis. In our study, 18.9% of the isolated *Enterococcus* spp. from birds carried this gene, especially *E. faecium*. Additionally, the *van*A and/or *van*B genes were identified in 48% of the isolates, with a higher prevalence of the *van*A gene, which is in agreement with the results obtained by Carvalho [20] and Guerrero-Ramos et al. [1] in Miranda donkey and wild game meat, respectively. It should be noted that most of the isolates that presented one or both of these genes belonged to the species *E. gallinarum* and *E. faecium*.

The *esp* gene, which was detected to a lesser extent in the study by Dolka et al. [31], was frequently detected in *Enterococcus* isolates in the present study, which agrees with the results of Iweriebor et al. [37]. In relation to the *gel*E gene, which was found to have the highest prevalence, most studies are in agreement [31,37].

Contrary to the studies performed by Carvalho [20] and Guerrero-Ramos et al. [1], in the present study *E. faecium* showed fewer genes of virulence, particularly the *cyl*L and *agg* genes. As in our study, Guerrero-Ramos et al. [1] also did not identify the *fsr* gene in any of the isolates.

In the present study, it was found that most of the isolates contained the *esp* gene, which is responsible for encoding the surface proteins that make the bacterial colony more persistent. Furthermore, this surface protein is implicated in cases of bacteremia, urinary tract infections and endocarditis [38]. The gene encoding cytolysin, *cyl*L, which was the most commonly detected in this study, is also associated with an increased risk of sudden death in cases of nosocomial bacteremia [39].

## 5. Conclusions

In conclusion, the results of this study suggest that ornamental pet foods found on the market may contain *Enterococcus* spp. with high levels of resistance to a variety of antibiotics and the ability to produce virulence factors. These foods can potentially carry epidemiologically important microorganisms and once ingested by ornamental animals can lead to the colonization of pathogenic *Enterococcus* spp. in the gut microbiota. Consequently, these bacteria, some of which are multidrug-resistant, can cause diseases in these animals with fatal consequences. Moreover, given the close contact with humans, they may cause infections and diseases, making antimicrobial treatment difficult.

Considering the limited information on the presence of multidrug-resistant enterococci in food intended to be fed to ornamental animals, this study highlights the need for greater control in all steps of food processing, as well as the potential risks to the environment and human and animal health. Additionally, this study contributes to the monitoring of antimicrobial resistance in a new, unexplored ecosystem niche, to facilitate the implementation of control measures.

## Figures and Tables

**Table 1:** Number of resistances observed from the antimicrobial susceptibility test of the 103 *Enterococcus* isolated from the ornamental animal feed.

Antibiotic Agent	Antimicrobial Class	Origin of Feed Sample			Total (%)
		Bird (*n* = 58)	Fish (*n* = 33)	Reptile (*n* = 12)	
Ampicilin	Penicilins	3	0	0	3 (2.9)
Vancomycin	Glycopeptides	1	2	0	3 (2.9)
Teicoplanin	Glycopeptides	1	2	0	3 (2.9)
Tetracycline	Tetracyclines	16	10	1	27 (26.2)
Erythromycin	Macrolides	29	18	3	50 (48.5)
Ciprofloxacin	Fluoroquinolones	24	8	7	39 (37.9)
Chloramphenicol	Phenicols	3	1	0	4 (3.9)
Quinupristin/ Dalfopristin *	Streptogramins	58	33	12	103 (100)
Nitrofurantoin	Nitrofurantoins	11	5	3	19 (18.4)
Rifampicin	Ansamycins	48	24	8	80 (77.7)
Fosfomycin	Fosfoycins	5	2	0	7 (6.8)
Gentamicin	Aminoglicosides	0	0	0	0 (0.0)
Streptomycin	Aminoglicosides	0	0	0	0 (0.0)
Linezolid	Oxazolidinones	15	3	2	20 (19.4)

*: intrinsic resistance.

**Table 2:** Antibiotic-resistant genes in *Enterococcus* species identified in this study (*n* = 60).

Antibiotic Gene	*E. faecalis* (*n* = 22)	*E. faecium* (*n* = 19)	*E. gallinarum* (*n* = 15)	*E. durans* (*n* = 4)	Total (%)
*erm*A *erm*B *erm*C	0 4 3	3 13 4	1 13 0	0 4 0	4 (6.7) 34 (56.7) 7 (11.7)
*tet*K *tet*M *tet*L	5 8 4	5 0 13	7 10 10	2 4 4	19 (31,7) 22 (36.7) 31 (51.7)
*aac*(6′′)-*aph*(2′′)	0	0	2	1	3 (5.0)
*cat*A	1	0	9	2	12 (20.0)
*vat*D *vat*E	1 4	0 2	1 4	2 1	4 (6.7) 11 (18.3)
*van*A *van*B	5 6	6 4	10 2	2 0	23 (38.3) 12 (20.0)
*ant*(6)-Ia	3	4	10	4	21 (35.0)

**Table 3:** Virulence genes detected in each *Enterococcus* species analyzed in this study (*n* = 60).

Virulence Gene	*E. faecalis* (*n* = 22)	*E. faecium* (*n* = 19)	*E. gallinarum* (*n* = 15)	*E. durans* (*n* = 4)	Total (%)
*esp*	10	0	12	2	24 (40.0)
*ace*	0	0	0	0	0 (0.0)
*gel*E	4	0	10	3	17 (28.3)
*agg*	6	2	7	3	18 (30.0)
*fsr*	0	0	0	0	0 (0.0)
*cpd*	3	0	8	1	12 (20.0)
*cyl*A	1	0	4	2	7 (11.7)
*cyl*B	2	0	1	0	3 (5.0)
*cyl*M	8	0	3	3	14 (23.3)
*cyl*L	8	6	13	4	31 (51.7)

**Table 4:** Significant associations obtained between resistance and virulence genes and the *Enterococcus* species.

		*E. faecalis*	*E. faecium*	*E. gallinarum*	χ^2^	*p*
Antibiotic resistance genes						
*erm*B	Positive Negative	4 18	13 6	13 2	19.37	0.0001
*tet*M	Positive Negative	8 14	0 19	10 5	17.38	0.0002
*tet*L	Positive Negative	4 18	13 6	10 5	13.10	0.0014
*van*A	Positive Negative	5 17	6 13	10 5	7.78	0.0205
*ant*6-Ia	Positive Negative	3 19	4 15	10 5	13.04	0.0015
Virulence factors genes						
*cyl*L	Positive Negative	8 14	6 13	13 2	12.27	0.0022

## Data Availability

The study did not report any data.

## Supplementary Materials

The following supporting information can be downloaded at: https://www.mdpi.com/article/10.3390/ani13111761/s1, Table S1: Total count of microorganisms in bird, fish, reptile and mammal food samples in colony forming unit per gram (CFU/g); Table S2: Phenotypic, genotypic and virulent profile of the 60 selected *Enterococcus* isolates based on food origin.
