# Supplementary material for: Antibiotic Resistance of Enterococcus Species in Ornamental Animal Feed
Source: Animals (Basel). 2023 May 26;13(11):1761. doi: 10.3390/ani13111761 (PMC10251925; doi:10.3390/ani13111761)
Supplement: Supplementary file 1 [file animals-13-01761-s001.zip › animals-2378864-supplementary.pdf]

animals

Supplementary material

Antibiotic Resistance of *Enterococcus* species in ornamental animal feed

Rúben Soares [1], Carla Miranda [1-3,†,*], Sandra Cunha [1], Luís Ferreira [1], Ângela Martins [4,5,6], Gilberto Igrejas [2,7,8] and Patrícia Poeta [1,2,5,6]

1 Microbiology and Antibiotic Resistance Team (MicroART), Department of Veterinary Sciences, University of Trás-os Montes e Alto Douro, 5000-801 Vila Real, Portugal; rubensoares297@gmail.com; sandra_mdc97@hotmail.com; luisferreira_12@hotmail.com; carlisabelmi@utad.pt; ppoeta@utad.pt
2 Associated Laboratory for Green Chemistry (LAQV-REQUIMTE), University NOVA of Lisbon, 1099-085 Caparica, Portugal;
3 Toxicology Research Unit (TOXRUN), University Institute of Health Sciences – CESPU (IUCS-CESPU), 4585-116 Gandra, Portugal;
4 Department of Zootechnics, University of Trás-os Montes e Alto Douro, Vila Real, Portugal; angela@utad.pt.
5 Veterinary and Animal Research Centre (CECAV), University of Trás-os Montes e Alto Douro, 5000-801 Vila Real, Portugal;
6 Veterinary and Animal Research Centre, Associate Laboratory for Animal and Veterinary Science (AL4AnimalS), Vila Real, Portugal.
7 Department of Genetics and Biotechnology, University of Trás-os-Montes e Alto Douro, 5000-801 Vila Real, Portugal; gigrejas@utad.pt
8 Functional Genomics and Proteomics Unit, University of Trás-os-Montes e Alto Douro, 5000-801 Vila Real, Portugal;
† These authors contributed equally to this work.
* Correspondence: carlisabelmi@utad.pt.

Citation: Soares, R.; Miranda, C.; Cunha, S.; Ferreira, L.; Martins, A.; Igrejas, G.; Poeta, P. Antibiotic Resistance of *Enterococcus* species in ornamental animal feed. *Animals* **2023**, *13*, x. https://doi.org/10.3390/xxxxx

Academic Editor: Firstname Lastname

Received: date
Accepted: date
Published: date

Publisher's Note: MDPI stays neutral with regard to jurisdictional claims in published maps and institutional affiliations.

Copyright: © 2023 by the authors. Submitted for possible open access publication under the terms and conditions of the Creative Commons Attribution (CC BY) license (https://creativecommons.org/licenses/by/4.0/).

Supplementary Materials: The following supporting information can be downloaded at: www.mdpi.com/xxx/s1, Table S1: Total count of microorganisms in bird, fish, reptile, and mammal food samples in colony forming unit per gram (CFU/g); Table S2: Phenotypic, genotypic and virulent profile of the 60 selected *Enterococcus* isolates based on food origin.

Table S1. Total count of microorganisms in bird, fish, reptile, and mammal food samples in colony forming unit per gram (CFU/g).

Birds		Fish		Reptiles		Mammals	
**Sample**	**CFU/g**	**Sample**	**CFU/g**	**Sample**	**CFU/g**	**Sample**	**CFU/g**
**A1**	30	**P1**	0	**R1**	20	**M1**	260
**A2**	0	**P2**	0	**R2**	800	**M2**	200
**A3**	250	**P3**	130	**R3**	2400		
**A4**	0	**P4**	100	**R4**	10		
**A5**	830	**P5**	10	**R5**	10000		
**A6**	600	**P6**	120	**R6**	100		
**A7**	320	**P7**	0	**R7**	0		
**A8**	3600	**P8**	20				
**A9**	27000	**P9**	100				
**A10**	70	**P10**	170				
**A11**	1100	**P11**	10				
**A12**	20	**P12**	60				
**A13**	110	**P13**	43000				
**A14**	50	**P14**	10				
**A15**	400	**P15**	10				
**A16**	60	**P16**	20				
**A17**	440	**P17**	20				
**A18**	1900	**P18**	10				
**A19**	10	**P19**	10000				
**A20**	200	**P20**	10				
**A21**	120	**P21**	20				
**A22**	0	**P22**	0				
**A23**	1800						
**A24**	3100						
**A25**	83000						
**A26**	0						

Table S2. Phenotypic, genotypic and virulent profile of the 60 selected *Enterococcus* isolates based on food origin.

Sample	Food origin	Species	Phenotypic profile	Genotypic profile	Virulent profile
P3(2)	Fish	*E. faecium*	RD	*erm*B-*tet*K-*tet*L-*van*A	-----
P3(4)	Fish	*E. faecalis*	RD	-----	*esp*-*cyl*M
P4(1)	Fish	*E. faecium*	RD	*erm*B-*tet*L	*cyl*L
P4(2)	Fish	*E. faecium*	TET-ERY-RD	*erm*B-*tet*L	*cyl*L
P3(V3)	Fish	*E. faecium*	VAN-TEC-F-FOS	*erm*B-*tet*K-*tet*L-*van*A	-----
P10(2)	Fish	*E. faecium*	CIP-RD	*erm*B-*tet*L	*cyl*L
P11(1)	Fish	*E. gallinarum*	TET-ERY-RD	*erm*B-*tet*M-*ant*(6)-*Ia*	*esp*-*gel*E-*cpd*-*cyl*L
P11(2)	Fish	*E. durans*	TET-ERY-RD	*erm*B-*tet*M-*tet*L-*vat*E-*van*A-*ant*(6)-*Ia*	*gel*E-*agg*-*cyl*M-*cyl*L
P12(1)	Fish	*E. durans*	TET-ERY-QD	*erm*B-*tet*M-*tet*L-*vat*D-*ant*(6)-*Ia*	*esp*-*gel*E-*agg*-*cyl*A-*cyl*M-*cyl*L
P12(2)	Fish	*E. durans*	TET-ERY-QD	*erm*B-*tet*K-*tet*M-*tet*L-*aac*(6′)-*aph*(2′′)-*Ia*-*cat*A-*van*A-*ant*(6)-*Ia*	*esp*-*gel*E-*agg*-*cpd*-*cyl*A-*cyl*M-*cyl*L
P12(4)	Fish	*E. gallinarum*	TET-ERY-QD	*erm*B-*tet*M-*tet*L-*cat*A-*van*A-*ant*(6)-*Ia*	*esp*-*gel*E-*agg*-*cpd*-*cyl*A-*cyl*M-*cyl*L
P13(2)	Fish	*E. faecalis*	ERY	*tet*M-*ant*(6)-*Ia*	*esp*-*gel*E-*agg*-*cpd*-*cyl*M-*cyl*L
P13(3)	Fish	*E. faecalis*	ERY	*tet*M	*esp*-*cpd*-*cyl*B-*cyl*M-*cyl*L
P19(4)	Fish	*E. faecium*	CIP-F-RD-LNZ	*erm*A-*erm*C-*vat*E-*van*B-*ant*(6)-*Ia*	-----
P20(2)	Fish	*E. faecalis*	CIP-F-RD	*van*B	-----
P20(3)	Fish	*E. faecalis*	TET-CIP-RD	-----	*cyl*L
P20(4)	Fish	*E. faecium*	TET-ERY-CIP-C-QD-RD	*van*B-*ant*(6)-*Ia*	*cyl*L
P21(1)	Fish	*E. faecalis*	CIP-RD-LNZ	-----	-----
P21(2)	Fish	*E. faecalis*	CIP-RD	*erm*B	-----
A3(1)	Bird	*E. faecium*	ERY- RD	*erm*B-*tet*L	*agg*
A5(1)	Bird	*E. faecium*	TET-ERY- RD	*erm*B-*tet*L	*cyl*L
A6(1)	Bird	*E. faecium*	---	*erm*B-*erm*C-*tet*L-*van*A-*ant*(6)-*Ia*	-----
A6(3)	Bird	*E. gallinarum*	TET-ERY	*erm*B-*tet*K-*tet*M-*cat*A-*van*A-*ant*(6)-*Ia*	*esp*-*gel*E-*cyl*L
A7(1)	Bird	*E. gallinarum*	RD	*erm*B-*tet*M-*tet*L-*ant*(6)-*Ia*	*esp*-*gel*E-*agg*-*cpd*-*cyl*B-*cyl*L
A7(2)	Bird	*E. gallinarum*	RD	*erm*B-*tet*k-*tet*L-*cat*A-*van*A-*ant*(6)-*Ia*	*esp*
A7(4)	Bird	*E. faecalis*	TET-RD	*tet*M-*ant*(6)-*Ia*	*esp*-*cyl*B-*cyl*M
A7(5)	Bird	*E. faecalis*	TET-RD	*tet*M	*cpd*-*cyl*M
A7(6)	Bird	*E. faecalis*	TET-RD	*tet*K	-----
A7(7)	Bird	*E. faecalis*	---	*vat*E	*esp*-*cyl*L
A7(8)	Bird	*E. faecalis*	---	*tet*L-*vat*E	*esp*-*cyl*L
A9(1)	Bird	*E. faecium*	RD	*erm*B-*erm*C-*tet*K-*tet*L-*van*A-*ant*(6)-*Ia*	-----
A9(2)	Bird	*E. durans*	---	*erm*B-*tet*K-*tet*M-*tet*L-*cat*A-*vat*D-*ant*(6)-*Ia*	*cyl*L
A9(3)	Bird	*E. faecalis*	---	*tet*M-*tet*L-*cat*A-*vat*D-*van*A-*ant*(6)-*Ia*	*esp*-*gel*E-*cyl*M-*cyl*L
A9(4)	Bird	*E. gallinarum*	---	*erm*B-*tet*K-*tet*M-*tet*L-*cat*A-*vat*D-*van*A	*esp*-*cyl*L
A10(1)	Bird	*E. faecium*	QD-RD-FOS	*erm*B-*tet*L-*van*A	*cyl*L

A10(2)	Bird	*E. gallinarum*	RD	*erm*B-*tet*M-*tet*L-*aac*(6′)-*aph*(2′′)-*Ia*-*cat*A-*vat*E-*van*A-*ant*(6)-*Ia*	*esp*-*gel*E-*agg*-*cpd*-*cyl*A-*cyl*L
A11(1)	Bird	*E. faecalis*	ERY-CIP-RD	-----	-----
A11(2)	Bird	*E. gallinarum*	TET-ERY- CIP-RD	*erm*B-*tet*K-*tet*M-*tet*L-*aac*(6′)-*aph*(2′′)-*Ia*-*ant*(6)-*Ia*	*esp*-*gel*E-*agg*-*cpd*-*cyl*M-*cyl*L
A13(1)	Bird	*E. faecium*	TET-ERY- RD	*erm*B-*tet*L	-----
A14(1)	Bird	*E. gallinarum*	QD-RD	*erm*B-*tet*M-*tet*L-*cat*A-*van*A-*ant*(6)-*Ia*	*esp*-*gel*E-*cyl*M-*cyl*L
A14(3)	Bird	*E. gallinarum*	CIP-QD-RD-LNZ	*erm*A-*vat*E-*van*A-*van*B	-----
A15(1)	Bird	*E. faecalis*	TET-ERY-CIP-RD-LNZ	*erm*C-*tet*K-*van*A-*van*B	*agg*
A15(3)	Bird	*E. gallinarum*	TET-ERY-CIP-F-RD-LNZ	*erm*B-*tet*L-*cat*A-*vat*E-*van*A	*gel*E-*agg*-*cyl*A-*cyl*L
A15(V1)	Bird	*E. faecalis*	ERY-CIP-F-RD	*erm*C-*van*A-*van*B	*agg*
A15(V3)	Bird	*E. faecalis*	AMP-TET-ERY-CIP-QD-F-RD-LNZ	ermC-tetK-vatE-vanA-vanB	*agg*
A15(V4)	Bird	*E. faecalis*	AMP-TET-ERY-CIP-QD-F-RD-FOS-LNZ	*tet*K-*van*A-*van*B	*agg*
A21(2)	Bird	*E. faecium*	ERY-CIP-C-QD-LNZ	*van*A-*van*B	*agg*
A23(2)	Bird	*E. gallinarum*	CIP-QD-F-RD	*erm*B-*tet*K-*tet*M-*tet*L-*van*B-*ant*(6)-*Ia*	*esp*-*cpd*-*cyl*A-*cyl*L
A24(1)	Bird	*E. faecium*	TET-CIP-QD-RD-LNZ	ermB-ermC-tetK-tetL	-----
A24(3)	Bird	*E. faecalis*	CIP-QD-F-RD-LNZ	*tet*M-*tet*L	*esp*-*cyl*L
A26(1)	Bird	*E. faecium*	AMP-TET-ERY-CIP-QD-RD-LNZ	*erm*B-*tet*K-*tel*L	-----
A26(2)	Bird	*E. faecalis*	TET-ERY-CIP-C-QD-RD-LNZ	*erm*B-*tet*M-*tet*L	*esp*-*gel*E-*cyl*M-*cyl*L
A26(3)	Bird	*E. faecalis*	TET-CIP-QD-RD-LNZ	*erm*B-*tet*K-*tet*M	*esp*-*gel*E-*agg*-*cyl*A-*cyl*M
R1(2)	Reptile	*E. gallinarum*	TET-ERY-CIP-QD-F-RD	*erm*B-*tet*K-*tet*L	*esp*-*cpd*-*cyl*L
R2(1)	Reptile	*E. gallinarum*	CIP-F-RD-LNZ	*cat*A-*vat*E-*van*A	*gel*E-*agg*-*cyl*L
R2(2)	Reptile	*E. faecium*	ERY-CIP-RD	*erm*A-*van*B	-----
R3(2)	Reptile	*E. gallinarum*	----	*erm*B-*tet*K-*tet*M-*cat*A-*van*A-*ant*(6)-*Ia*	*esp*-*gel*E-*agg*-*cpd*-*cyl*L
R6(1)	Reptile	*E. faecalis*	CIP-RD	*erm*B-*vat*E-*van*B	-----
R6(2)	Reptile	*E. faecium*	CIP-RD	*vat*E	-----
R6(4)	Reptile	*E. faecium*	CIP-RD	-----	-----
